# Active Surveillance in Prostate Cancer: Role of Available Biomarkers in Daily Practice

**DOI:** 10.3390/ijms22126266

**Published:** 2021-06-10

**Authors:** Belén Pastor-Navarro, José Rubio-Briones, Ángel Borque-Fernando, Luis M. Esteban, Jose Luis Dominguez-Escrig, José Antonio López-Guerrero

**Affiliations:** 1 Laboratory of Molecular Biology, Fundación Instituto Valenciano de Oncología (IVO), 46009 Valencia, Spain; bpastor@fivo.org; 2 Príncipe Felipe Research Center (CIPF), IVO-CIPF Joint Research Unit of Cancer, 46012 Valencia, Spain; 3 Department of Urology, Fundación Instituto Valenciano de Oncología (IVO), 46009 Valencia, Spain; jrubio@fivo.org (J.R.-B.); jldominguezescrig@hotmail.com (J.L.D.-E.); 4 Department of Urology, University Hospital Miguel Servet, IIS-Aragón, 50009 Zaragoza, Spain; aborque@comz.org; 5 Department of Applied Mathematics, Engineering School of La Almunia, University of Zaragoza, 50100 Zaragoza, Spain; lmeste@unizar.es; 6 Department of Pathology, School of Medicine, Catholic University of Valencia ‘San Vicente Martir’, 46001 Valencia, Spain

**Keywords:** prostate cancer, risk predictors, active surveillance, clinical biomarkers

## Abstract

Prostate cancer (PCa) is the most commonly diagnosed cancer in men. The diagnosis is currently based on PSA levels, which are associated with overdiagnosis and overtreatment. Moreover, most PCas are localized tumours; hence, many patients with low-/very low-risk PCa could benefit from active surveillance (AS) programs instead of more aggressive, active treatments. Heterogeneity within inclusion criteria and follow-up strategies are the main controversial issues that AS presently faces. Many biomarkers are currently under investigation in this setting; however, none has yet demonstrated enough diagnostic ability as an independent predictor of pathological or clinical progression. This work aims to review the currently available literature on tissue, blood and urine biomarkers validated in clinical practice for the management of AS patients.

## 1. Introduction

In 2020, 19.3 million new cases of cancer were diagnosed worldwide, according to the International Agency for Research of Cancer, of which 1,414,259 were of prostate cancer (PCa). These figures place PCa in the fourth position in incidence when combining both sexes, preceded by breast, lung and colorectum cancer [1]. When only men are considered, PCa represents the second most frequent malignancy after lung cancer (14.1% and 14.3%, respectively), being considered a global health problem, due to both the volume of population affected and its economic impact on Health Systems. 

Almost 90% of new PCa are localized and they are clinically classified in three risk groups, based on the serum prostate-specific antigen (PSA) levels, local clinical stage and histological aggressiveness provided by the Gleason Score (GS): high risk, intermediate risk, low risk and very low risk [2]. All clinical guidelines, such as European Society of Medical Oncology (ESMO) [3], National Comprehensive Cancer Network (NCCN) [4] and 2020 EAU-EANM-ESTRO-ESUR-SIOG [5], follow this classification with very little variation.

A recent study, which aimed to characterize PCa features, identified five clinical risk groups: low-risk localised (LRL), intermediate-risk localised (IRL), high-risk localised (HRL), positive nodal (N+) and metastatic (M1). Among 635,733 PCa cases analysed, the overall risk group distribution at diagnosis was: LRL 10.5% (*n* = 66,959), IRL 49.7% (*n* = 316,227), HRL 34.8% (*n* = 221 494), N+ 1.5% (*n* = 9150) and M1 3.5% (21,903). Furthermore, the proportions of patients who died from PCa in the five groups were 3.2%, 5.1%, 10.2%, 19% and 57.2%, respectively [6]

Before active surveillance (AS) appeared, the conventional therapeutic approach was to offer active treatment, either with surgery or radiotherapy, achieving high cure rates. However, they are not exempt from side effects at 5 years, including urinary incontinence, erectile dysfunction and bowel toxicity [7]. 

PCa occupies the fifth position in terms of mortality, with 375,304 cases in 2020 worldwide, preceded by lung, liver, stomach and colorectal cancers [1]. This difference between incidence and mortality is due to both the favourable impact of the treatments and the heterogeneous aggressiveness of these tumours. Furthermore, PCa has a slow evolution, even latent, and most tumours are diagnosed well before they are considered as clinically significant PCa (csPCa), characterized by International Society of Urological Pathology (ISUP) histopathology grade ≥2 and/or volume ≥0.5 cc and/or extraprostatic extension [8]. In fact, one-third of screening-detected PCa never becomes a health problem. Reinforcing this evidence, it is well known that 59% of necropsies of men over 79 years of age who died from other causes have latent PCa [9].

The effects of overdiagnosis, a consequence of current diagnostic pathways, could be minimized by progressive improvements in the characterization of indolent PCa, as well as, the implementation of less aggressive therapeutic strategies such as AS, with promising results after long follow-up, of up to 8 years [10].

In only a decade, AS has moved from uncertainty and low acceptability among urologists to representing the first therapeutic option in every clinical guideline [4,5] for very low/low risk and even some de novo intermediate-risk tumours [3,11]. However, the trend today is that AS protocols should pursue the reduction of strict follow-up biopsy schemes [12], and personalised biopsy schedules have been proposed based on the risk of Gleason upgrading. This reduction could also be favoured by improving the selection of low-risk PCa both with biomarkers or multiparametric magnetic resonance imaging (mpMRI) [13].

The new taxonomic classification of PCa in seven molecular biotypes has given some light to the biologic and molecular characterization of such a heterogeneous disease, but it is still far from changing daily practice [14]. AS has evolved with the refinement of imaging, where multiparametric magnetic resonance imaging (mpMRI) has been key, but it is still limited when used alone for clinical decision-making, together with its availability, cost and dependency on the radiologists experience as collateral drawbacks [15,16]. We have focused on commercially available biomarkers for AS management in our review, although we recognize that mpMRI is already an established tool that has optimized AS management; hence, potential synergies between mpMRI and some biomarkers have also been addressed.

As mentioned above, biomarkers would constitute our second tool. They should “scan” the whole prostate and be widely available, reproducible, inexpensive, objective and with adjusted predictions. However, these are characteristics that not all biomarkers demonstrate, and the search continues to find the “ideal biomarker”, the “Holy Grail” studying serum, urine or prostatic tissue [17,18]. Knowing that the molecular classification of PCa will probably pave the road for more specific biomarkers in different molecular biotypes, the aim of this study is to review the present literature in clinically available biomarkers that could help to optimize AS in daily practice.

## 2. Evidence Acquisition

A comprehensive literature search from January 2013 to March 2021 was performed in PubMed, including articles written in English language, reporting on PCa diagnosis and follow-up biomarkers. A specific search strategy was designed combining the following keywords: “prostate cancer”, “active surveillance”, “tissue biomarkers”, “blood biomarkers”, “urine biomarkers” and “clinical significance”. In particular, the following search blocks were used for the PubMed database (active surveillance) AND (prostate cancer) AND (biomarker) AND [(tissue) OR (blood) OR (urine)] AND (clinical significance). Due to the exigent search criteria used, mainly based on the addition of the “clinical significance” label, secondary sources were also examined in a descriptive manner. Cross-referenced potentiality relevant articles, not identified in the primary search, were also considered and hand-picked. Case reports, editorials, letters, congress abstract and congress communications were not eligible.

Original research articles were curated based on favouring large sample sizes, independent validation and patients directly included in AS programs and not just as per its role in optimizing PCa diagnosis. When biomarkers were studied in diagnosis, we focused our interest in those cohorts already in AS and its role in its clinical management. All abstracts were reviewed by expert urologists (J.R.-B., A.B.-F., J.L.D.-E.).

**Figure 1:**
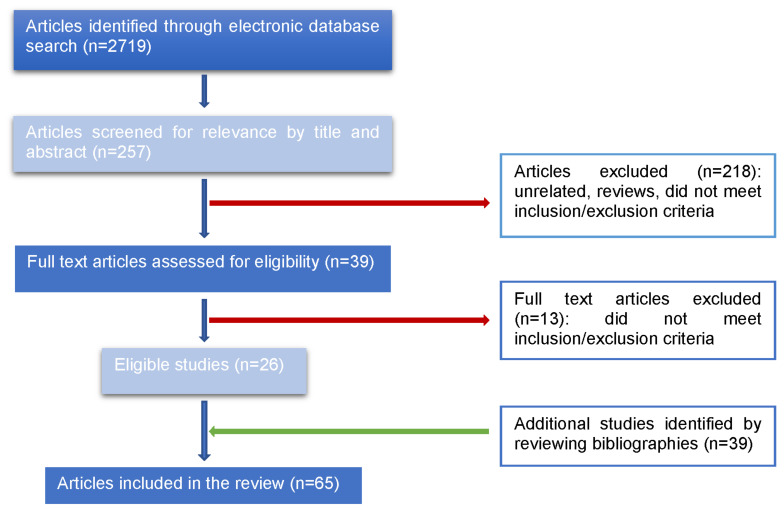
Workflow diagram of the references’ selection process. Bibliography was selected considering different sources of biomarkers including tissue, blood and urine.

After exclusion of duplicates and articles unrelated to the topic of this review, 267 full-text records were screened, and finally, 26 papers accomplished final eligibility. Then, 39 other papers were added that focus on commercially available markers related to the topic. The article selection process is shown in a workflow diagram (Figure 1). 

## 3. Evidence Synthesis

### 3.1. Blood Biomarkers

○ PSA

Nobody questions the role of PSA in PCa screening, with the latest publication of the European Randomized Screening in Prostate Cancer setting its irreplaceable importance [19]. However, once diagnosed, in those PCa candidates for AS, its role as biomarker has been questioned due to its variability and has been preferably studied related to its changes with time, prostate volume or other isoforms.

○ PSA kinetics

Diagnostic and follow-up of PCa patients based on PSA measurements are still the most common strategies, although PSA has been demonstrated as an unspecific biomarker in the AS setting. The rate of PSA change over time, known as PSA dynamics or kinetics (PSAk), was conceptually introduced by Carter in 1992 [20]. PSAk has been shown to overcome PSA limitations and to predict PCa reclassification in men enrolled in AS programs. 

Cooperberg et al., calculated PSAk using a linear mixed-effect model, in which the natural logarithm of PSA (ln[PSA]) was modelled as a linear function of time since the diagnosis, with a random intercept indicating the individual-specific ln[PSA] at diagnosis and a random slope reflecting the individual-specific rate of change over time. It was tested in a multicentre cohort with long-term follow-up, suggesting that collecting PSA measurements over time could be clinically useful at predicting outcomes in men with PCa on AS [21]. 

Related to PSAk, PSA doubling-time (PSADT) is the number of years over which a certain level of PSA increases by a factor of two and is calculated as DT = ln(2)/m, where m is the slope of the regression of ln[PSA] over time [22]. In the prospective multicentre Canary Prostate Active Surveillance Study (PASS), PSADT < 36 months was originally a criterion for progression, and some authors recommended this parameter for the detection of aggressive tumours during AS [23]. However, it was found to be unspecific [24] and must be evaluated as a part of the PSAk linear model.

On the other hand, PSA velocity (PSAV) represents a change in PSA level over time and is calculated by linear regression of untransformed PSA values. By calculating the number of times that serial PSAV measurements pass a threshold, Patel et al. developed the PSAV Risk Count (PSAV RC) score. When used in a cohort of very low-risk PCa, it was associated with an increased risk of biopsy reclassification due to any unfavourable pathology finding; hence, PSAV RC is proposed for monitoring patients on AS and to decrease the frequency of biopsies in the long term [25]. 

In summary, higher PSAV and shorter PSADT kinetics might be useful in differentiating between PCa with more aggressive potential. The association between pre-treatment PSAk and PCa biology is supported by multiple studies that found a strong association between the PSAV and PSADT and various pathological features of aggressive PCa [22].

○ PSA density (PSAD)

The PSA density (PSAD), calculated by dividing the preoperative PSA by the prostate volume (without seminal vesicles), was introduced in the early 1990s by Benson et al. [26]. Its role in predicting upgrading and reclassification in men with low-risk PCa enrolled in AS has been assessed in several studies. 

A study recently published by Yusim et al. including 992 men with a median age of 66 years concluded that patients with PSAD higher than 0.34 ng/mL^2^ have a 56.4% chance of being diagnosed with a csPCa, with risks estimated at 4%, 8.5% and 31.5%, for PSADs <0.09 ng/mL^2^, between 0.09 and 0.19 ng/mL^2^ and between 0.19 and 0.34 ng/mL^2^, respectively [27]. Moreover, it has been demonstrated that PSAD values differ between African American (AA) men and Caucasian men, being lower in AA patients in cases of similar tumour volumes [28]. 

In summary, PSAD is proposed to be tested prior to prostate biopsy as it is an inexpensive and widely available tool, which may avoid unnecessary biopsies. Its inclusion in AS protocols could improve inclusion criteria and follow-up of PCa patients. Furthermore, the more accurate prostate volume calculation by MRI might potentially make its use more reliable [27,29].

○ Prostate Health Index (PHI)

PHI, from Beckman Coulter [30], is a diagnostic blood test that combines free (fPSA), total PSA (tPSA) and isoform [-2]proPSA into a single score, calculated according to the formula [-2]proPSA/fPSA x √tPSA, developed to maximize specificity at high sensitivity [31]. Approved by the USA Food and Drug Administration (FDA), it is now considered by the NCCN guidelines in the diagnostic setting [4]. 

Chiu et al., demonstrated that PHI and %p2PSA are predictors of RP pathologic outcomes such as pT3, pGS, GS upgrade, tumour volume >0.5 mL, and Epstein criteria for significant tumours [32]. Moreover, Hirama et al. showed the high diagnostic ability of p2PSA-related parameters, such as %p2PSA and PHI, for discriminating patients with non-significant cancer from those with significant cancer, being useful markers for AS [23]. 

Several studies compared the predicting value of PHI and PCA3. Cantiello et al., found a superior predictive accuracy of PHI over PCA3 (AUC 0.92 vs. AUC 0.77) in discriminating clinically significant disease in men eligible for AS, outperforming PCA3 performance, resulting in a higher net benefit [33,34]. 

The use of these markers may reduce the incidence of underestimation at initial diagnosis, enabling a more accurate selection of candidates suitable for AS. PHI measurement could be clinically useful in discriminating the presence of insignificant PCa in AS candidates [23,31,32,33]. Furthermore, its utility is increased by adding mpMRI to discriminate the presence of pathologically confirmed significant PCa, as demonstrated in a cohort of patients who underwent RP but were eligible for AS [35].

○ 4Kscore^®^

The 4Kscore^®^ is a prebiopsy blood test developed by OPKOlab that incorporates a panel of four kallikreins (tPSA, fPSA, intact PSA [iPSA] and human kallikrein 2 [hK2]) combined with clinical features such as the 4Kscore^®^, which determines a man’s risk for high-grade aggressive PCa (GS ≥ 7) [36], improving decision making regarding prostate biopsy. 

The 4Kscore^®^ provides a score ranging from 0 to100%, reflecting the probability of finding a significant PCa at biopsy; hence, 100% minus the 4Kscore result is the personalized negative predictive value (NPV) or probability that a patient will not have Gleason ≥7 cancer on prostate biopsy [37]. 

Lin et al. evaluated the 4Kscore^®^ in a cohort of patients candidates for AS, already diagnosed with cancer, demonstrating that the addition of 4Kscore^®^ to a model containing clinical information significantly improves the prediction of the outcome in the first surveillance biopsy, which is associated with reclassification [38]. 

Similarly, in a prospective evaluation by Borque-Fernando et al. in an AS scenario, concluded that the 4Kscore^®^, at a cut-off 7.5%, was significantly associated with tumour reclassification at the confirmatory biopsy, while the previously used %fPSA/tPSA ratio did not show this association [39]. 

Due to the ability of the 4Kscore^®^ to discriminate between men who are likely to harbour clinically relevant PCa and those with indolent tumours or no cancer, it has been proposed as a good marker to identify patients who are more likely to benefit from biopsy, due to a higher risk of csPCa requiring active treatment [39,40] potentially implemented in an AS setting [37,38,39,40,41].

### 3.2. Tissue Biomarkers

○ Oncotype DX^®^ Genomic Prostate Score (GPS)

The Oncotype Dx^®^ Genomic Prostate Score (GPS), from Genomic Health^®^ [42], is an RNA based expression assay that includes 12 PCa related genes normalized to five housekeeping genes, which can be performed on needle core biopsy tissues. This test uses real-time quantitative polymerase chain reaction (RT-qPCR) to measure expression levels of genes related to four tumour aggressiveness pathways, including androgen signalling (*AZGP1*, *KLK2*, *SRD5A2*, and *FAM13C*), cellular organization (*FLNC*, *GSN*, *TPM2*, and *GSTM2*), stromal response (*BGN*, *COL1A1*, and *SFRP4*) and cellular proliferation (*TPX2*) [43]. 

GPS has been shown to predict adverse surgical pathology (AP) and biochemical recurrence (BR) in men diagnosed with low- and intermediate-risk PCa treated with immediate surgery [44,45]. It has been used as a tool to inform the decision making of immediate treatment versus AS in men newly diagnosed with low- or favourable intermediate-risk PCa and was recently included in the NCCN guidelines [46]. Some studies have demonstrated that GPS is associated with an increased risk of AP findings in AS patients who later underwent radical prostatectomy (RP), being also associated with BR following surgery in such patients [46,47]. The GPS ranges from 0 to 100, with higher scores indicating a greater risk of aggressive disease. 

Eure et al. reported higher rates of AS uptake (62% vs. 40%) and persistence on AS at 1 year (55% vs. 35%) among men who underwent the GPS testing compared to those who did not [48]. The GPS test has been recommended in men with early-stage PCa [49]. In this way, after obtaining a GPS, more patients were re-classified as very low-risk compared to the initial distribution determined by the NCCN risk category alone [42]. Regarding reproducibility, the initial test is the most informative, and serial testing seems to have limited benefit [43]. 

Although higher GPS has been associated with an increased risk of AP and BR, it is reasonable to use the GPS in conjunction with other known clinical risk factors when selecting patient candidates for AS [47].

○ Genome DX Decipher^®^ Genomic Classifier

Decipher^®^ Prostate Cancer Test, from Genome Dx Biosciences [50], is a 22-feature RNA biomarker assay that has been developed to predict metastasis risk at 5- and 10-years after RP [51]. This test incorporates 22 coding and non-coding genes that cover seven cancer pathways, including angiogenesis, invasion and metastasis, or growth and differentiation [50]. It generates a score ranging from 0 to 1, with higher values indicating an increased probability for both AP and poorer oncologic outcomes [52].

Decipher^®^ is a significant predictor of AP when used alone or combined with clinical risk stratification systems [53], and when applied to prostatectomy tissue from NCCN very low-/low- and favourable intermediate-risk patients, it has identified patients inappropriately selected for AS. 

It has been demonstrated that Decipher^®^ at the biopsy cores outperformed NCCN clinical risk grouping, biopsy GS and preoperative PSA; for every 10% increase in the Decipher^®^ score, the high-risk increased by 1.72 [53]. Notably, combining Decipher^®^ with NCCN risk groups increased the concordance index (C-index) from 0.75 to 0.88, suggesting that Decipher^®^ captures a considerable proportion of PCa available on diagnostic needle biopsies obtained in routine clinical practice. Decipher^®^ scores on biopsies were also predictive of other key outcomes, including the presence of primary Gleason pattern 4/5 and an elevated risk of metastasis within 5 years [54]. Applying the Decipher ^®^ test appears to better guide treatment recommendations [55].

○ Prolaris^®^ (Cell Cycle Progression Score)

The Cell Cycle Progression (CCP) score, from Myriad [56], is a validated prognostic RNA expression signature, based on the expression levels of 31 genes related to cell cycle progression and 15 housekeeping genes. Several studies have demonstrated that the CCP score is strongly associated with progression to metastatic disease after both surgery and radiotherapy [57,58,59]. 

The CCP score threshold was developed in men who might typically be considered for AS based on having low- or favourable intermediate-risk disease according to a conservative interpretation of NCCN guidelines [46]. The combination of CCP score with clinico-pathologic risk factors from the Cancer of the Prostate Risk Assessment (CAPRA) model [60] results in the cell cycle risk (CCR) test, with a better classification performance. The CCR-based risk stratification might guide the identification of patient candidates to AS from those who would require more active intervention [58]. Both the molecular score and the derived predicted risk might be used in both AA and non-AA patients with newly diagnosed PCa [59].

Lin et al., validated this test in conservatively managed men and showed that the CCR score threshold significantly dichotomized men with high-risk (CCR >0.8) and low-risk (≤0.8) of 10-year PCa mortality, indicating that the threshold can be safely used to identify candidates for AS. Application of the CCR score threshold should result in a substantial increase in men who would be considered candidates for AS that would have traditionally been excluded [57].

### 3.3. Urine Biomarkers

○ Prostate Cancer Antigen 3 (*PCA3*)

*PCA3* was first described in 1999 by Bussemakers et al. [61] as a non-coding mRNA only expressed in human prostate tissue but significantly overexpressed in PCa and is used in clinical practice as Progensa^®^ *PCA3* assay, an in vitro nucleic acid amplification test, developed by Hologic [62], approved by the FDA to be used in conjunction with other patient information to aid the decision for repeat biopsy in men 50 years of age or older with one or more previous negative prostate biopsies. 

This *PCA3* score is generated as a ratio of *PCA3* mRNA to PSA mRNA in urine multiplied by 1000, and a *PCA3* score <25 is associated with a decreased likelihood of positive biopsy [63,64].

Tosoian et al., evaluated it in a cohort of men with favourable-risk PCa included in AS programs and who underwent tumour reclassification, demonstrating that patients who reclassified had significantly higher *PCA3* scores at both initial (48.0 vs. 24.5, *p* = 0.007) and subsequent (63.5 vs. 36.0, *p* = 0.002) measurements, indicating an association between *PCA3* and grade reclassification [65]. 

However, despite the demonstrated *PCA3* utility for PCa diagnosis, several studies suggest that additional biomarkers should be incorporated into *PCA3* to ensure detection of high grade [66]. 

The addition of mpMRI to *PCA3* reduces overdetection and overtreatment of indolent PCa by improving diagnostic accuracy. Hence, the use of urinary *PCA3* testing in men with low or equivocal suspicion mpMRI allows unnecessary biopsies, the refinement of risk stratification and the optimization of high-grade cancer detection to be reduced [64].

○ TMPRSS2:ERG (T2E) fusion gene

The *T2E* fusion gene was first discovered in 2005 by Tomlins et al. [67], and it is present in approximately 50% of PCas. It constitutes a highly specific biomarker that can be detected both in FFPE and urine samples [68,69]. 

In a prospective study by Lin et al., post-digital rectal examination (post-DRE) urine samples were collected and T2E levels were analysed, indicating an association with higher tumour volume and higher GS in subsequent biopsies [70]. Furthermore, high *T2E* expression levels have been associated with an increased risk of tumour upgrading and upstaging in AS candidates [71]. 

In an AS context, Whelan et al., assessing the effectiveness of *T2E* expression in prostatic secretions, showed that non-invasive *T2E* measurement in urine may refine patient acceptance into AS programs [72]. 

However, a comparative study between urinary *PCA3* and *T2E* measures during multiple times at surveillance found that these markers add little or no improvement in clinical variables in predicting biopsy reclassification due to the high PCa heterogeneity [73,74]. Hence, both *PCA3* and *T2E* are proposed to be combined in a risk calculator, MiProstate Score (MiPS), which shows higher specificity and sensitivity [75,76].

○ SelectMDx

Related to urinary biomarkers useful in predicting csPCa, a novel urinary assay-based risk score called SelectMDx from MDXHealth^®^ was developed by combining serum PSA, PSAD and clinical factors such as age and prior negative biopsy with two mRNA signatures, namely urinary homeobox C6 (*HOXC6*) and distal-less homeobox 1 (*DLX1*), and *KLK3* gene as a reference [77]. 

Leyten et al. developed the test based on detecting increased mRNA levels *HOXC6*, *DLX* and tudor-domain-containing 1 (*TDRD1*), which have been selected between eight candidates because of having independent additional predictive value of PSA for the detection of biopsy GS ≥7. These genes have been associated with PCa development [78]. In this study, it was shown that the combination of urinary *HOXC6* and *DLX1* was superior to Progensa^®^ *PCA3* in the diagnosis of GS ≥ 7 PCa. 

Based on the urinary biomarker panel previously defined, Van Neste et al. proposed a new test by combining *HOXC6* and *DLX1* mRNA expression levels with traditional clinical risk factors, such as PSAD, DRE, PSA, age, history of prostate biopsy and family history, which is able to detect high-grade csPCa accurately and consequently could be used in AS decision making, reducing unnecessary biopsies and potential overtreatment [79]. 

We have just published our results with SelectMDx and *PCA3* in an AS setting, showing that SelectMDx showed statistically significant differences related to pathological progression-free survival (HR: 1.035; 95% CI: 1.012–1.057) (*p* = 0.002) with a C-index of 0.670 (95% CI: 0.529–0.810) and AUC of 0.714(95% CI: 0.603–0.825) at 5 years. The combination of both biomarkers did not improve the prediction of PP and C-index 0.630 (95% CI: 0.455–0.805) [80]. Despite the high ability of SelectMDx in predicting the likelihood of finding high-grade PCa, it has been demonstrated that its combination with mpMRI could better select candidates to AS, identifying men who harbour csPCa, hence improving the cost-effectiveness. Pepe P et al., found that mpMRI and SelectMDx missed 3/9 (33.3%) and 4/9 (44.5%) of csPCa, respectively. Moreover, mpMRI combined with SelectMDx diagnosed 7/9 (77.8%) csPCa, outperforming SelectMDx alone, in a cohort of men enrolled in an AS protocol [81,82]. Nonetheless, several studies have demonstrated the potential cost-effectiveness of SelectMDx alone and instead of using mpMRI. In Spain, the cost-saving result of using the SelectMDx strategy has been estimated at EUR 247 per patient, which is EUR 20 million per yearly cohort, mostly by preventing the detection of insignificant cancers and, to a lesser extent, by reducing the number of biopsies [83]. Similarly, Dijkstra et al. showed that SelectMDx could improve PCa patients’ quality of life and detect high-grade tumours while saving cost compared to the current standard of care [84]. 

## 4. Conclusions

Active Surveillance (AS) is increasingly used in all health systems, balancing screening benefits with lowering overtreatment in PCa. Since this strategy was introduced, AS has evolved from initial protocols a decade ago, allowing inclusion without any reliable imaging, to more refined contemporary management pathways, guided by mpMRI imaging and fusion biopsy. Nevertheless, mpMRI is not widely available everywhere, and it is still very dependent on radiologist expertise and hence lacks homogeneity.

Biomarkers might play a role, probably combined with mpMRI and mainly in PIRADs 3 lesions, the “grey box”, where results are unclear. Their potential to give information from the scan the whole gland, their homogeneity, reproducibility and potential for comparison make them very attractive in the AS setting, but their prices should come down in order to be generalized, mainly tissular, markers. Most of them have shown in pilot studies their complementary role with mpMRI, but none is going to replace it when a biopsy is needed, at both the confirmatory and follow-up phases.

The main limitation that we have found in this review is that there are few studies specifically focused on applying these biomarkers in AS series. However, given the applicability that many of these tests have in identifying csPCa, they are good candidates for being tested in an AS scenario (Table 1). 

Tissular biomarkers, mainly those analysing independent molecular markers not related to classical clinic-pathological variables, could have a role in the future of AS management. However, issues in terms of high costs and difficulties in handling are drawbacks that should overcome before widespread implementation is possible. Therefore, at present, we can just recommend them in doubtful cases such as high-volume Gleason 3 + 3 or favourable intermediate-risk PCa where a more conservative management could be considered in case of favourable tissular marker scoring.

Prospective and longitudinal studies with different biomarkers focused on AS cohorts are still missing. Prospective, well-designed studies assessing predefined clinically relevant endpoints are needed to fully assess the real potential of these biomarkers and to compare them with other diagnostic tools, such as novel predictors of grade progression and mpMRI- PRECISE criteria [85]. Robust clinical evidence derived from such studies will support and guide clinical decision-making in the selection, management and long-term follow-up of PCa patients in AS schemes. 

## Figures and Tables

**Table 1:** Summary of clinically available biomarkers and tests with AS application.

Biomarker	Source	Characteristics	AS Application	References
**PSA**	Blood	PSA is a glycoprotein secreted by prostatic epithelial cells that lyses the clotted ejaculate to enhance sperm motility. It is the most important commonly used biomarker, but it has been shown not to be a cancer-specific marker, as some prostate diseases could also produce PSA elevated levels	Its role as biomarker in AS has been questioned due to its variability and has been mostly studied related to its changes with time, prostate volume or other isoforms	[19]
**PSA kinetics**	Blood	Rate of PSA change over time: PSA doubling time (PSADT) is the number of years over which a certain level of PSA increases by a factor of two and is calculated as DT = ln(2); PSA velocity (PSAV) represents a change in PSA level over time/m	Differentiates between PCa with more and less aggressive natural history	[20,21,22,23,24,25]
**PSA density (PSAD)**	Blood	Dividing preoperative PSA by prostate weight without seminal vesicles	Predicts upgrading and reclassification in men with low-risk PCa enrolled in AS, by improving the inclusion criteria and follow-up of PCa patients	[26,27,28,29]
**Prostate Health Index (PHI)**	Blood	Index calculated as [-2]proPSA/fPSA x √PSA	PHI measurement could be clinically useful in discriminating the presence of insignificant PCa in active surveillance candidates	[30,31,32,33,34,35]
**4Kscore^®^**	Blood	A panel of four kallikreins (tPSA, fPSA, intact PSA [iPSA], and human kallikrein 2 [hK2]) combined with clinical data available before cancer diagnosis	Identifies patients most likely to benefit from biopsy because of a high risk of having a clinically significant tumour that would require active treatment	[36,37,38,39,40,41]
**Oncotype DX^®^ Genomic Prostate Score (GPS)**	Tissue	RNA based expression assay of 12 PCa related normalized to 5 housekeeping genes	Associated with an increased risk of AP and BR in the initial test of early-stage PCa	[42,43,44,45,46,47,48,49,50]
**Genome DX Decipher^®^ Genomic Classifier**	Tissue	Includes 22 coding and non-coding genes, which covers seven cancer pathways, such as angiogenesis, invasion and metastasis, or growth and differentiation	Helps to predict metastasis risk after RP and exclude patients of following AS programs	[51,52,53,54,55,56]
**Prolaris^®^ (Cell Cycle Progression Score)**	Tissue	RNA expression signature based on measuring the expression levels of 31 genes that participate in cell cycle progression and 15 housekeeping genes	Helps to identify patients who may warrant increased intervention intensity due to their predicted risk of metastatic disease	[57,58,59,60,61]
**Prostate Cancer Antigen 3 (*PCA3*)**	Urine	Non-coding mRNA only expressed in human prostate tissue but overexpressed in PCa tissue	Patients who reclassified had significantly higher PCA3 scores at both initial and subsequent measures, indicating an association with grade reclassification	[62,63,64,65,66,67]
***TMPRSS2:ERG* fusion gene**	Urine	*T2E* fusion gene is present in approximately 50% of prostatic tumours and is detected both in FFPE and urine samples	Non-invasive *T2E* measurement in urine may refine patient acceptance into AS programs	[68,69,70,71,72,73,74,75,76,77]
**SelectMDx**	Urine	Combines serum PSA, PSAD and clinical factors such as age and prior negative biopsy with mRNA signatures: urinary homeobox C6 (HOXC6) and distal-less homeobox 1 (DLX1)	Can detect high-grade csPCa accurately and could therefore be used in AS decision making, reducing the number of unnecessary prostate biopsies and potential overtreatment	[78,79,80,81,82,83,84,85]

## Data Availability

Not applicable.

## Abbreviations

AA	African American
AP	adverse surgical pathology
AS	active surveillance
AUC	area under the curve
BR	biochemical recurrence
CAPRA	Cancer of the Prostate Risk Assessment
CCP	Cell Cycle Progression
CCR	cell cycle risk
C-index	concordance-index
CsPCa	clinically significant prostate cancer
*DLX1*	distal-less homeobox 1
DRE	digital rectal examination
ESMO	European Society of Medical Oncology
FDA	Food and Drug Administration
FFPE	formalin-fixed paraffin-embedded
fPSA	free PSA
GPS	Genomic Prostate Score
GS	Gleason score
*HOXC6*	urinary homeobox C6
HRL	high-risk localised
iPSA	intact PSA
IRL	intermediate-risk localised
ISUP	International Society of Urological Pathology
ln(PSA)	logarithm of PSA
LRL	low-risk localised
M1	metastatic
MiPS	MiProstate Score
mpMRI	multiparametric magnetic resonance imaging
N+	nodal positive
NCCN	National Comprehensive Cancer Network
PASS	Canary Prostate Active Surveillance Study
PCa	prostate cancer
PCA3	prostate cancer antigen 3
PCPTRC	Prostate Cancer Prevention Trial risk calculator
PHI	Prostate Health Index
PSA	prostate-specific antigen
PSAD	prostate-specific antigen density
PSADT	prostate-specific antigen doubling-time
PSAk	prostate-specific antigen kinetics
PSAV	prostate-specific antigen velocity
PSAV RC	PSAV Risk Count
RT-qPCR	real-time quantitative polymerase chain reaction
*T2E*	TMPRSS2:ERG
*TDRD1*	tudor domain containing 1
tPSA	total PSA
